# **Erythema Multiforme as a Result of Orf Disease; a Case Report**

**Published:** 2016

**Authors:** Tahmine Biazar, Mehran shokri, Hajar Hosseinnia, Masomeh Bayani

**Affiliations:** 1 Infectious Diseases and Tropical Medicine Research Center, Babol University of Medical Sciences, Babol, Iran.; 2 Clinical Research Development Unit, Rouhani Hospital, Babol University of Medical Science. Babol, Iran.

**Keywords:** Orf virus, erythema multiforme, emergency department, infectious disease medicine

## Abstract

Orf is a mucocutaneous disease that occurs when non-intact skin comes into contact with contaminated sheep saliva. The lesions may complicate to lymphangitis or secondary bacterial infection, but systemic complications such as erythema multiforme, maculopapular rash, and generalized lymphadenopathy are rare. In this paper, we present two cases of erythema multiforme following Orf disease.

## Introduction:

Orf is a mucocutaneous disease caused by double-stranded DNA parapoxviruses that is also known as sheep pox, ecthyma contagiosum, and contagious pustular dermatitis (-). Human transmission occurs when non-intact skin comes into contact with contaminated sheep and goat saliva and their dead body (4). The incubation period varies from 3 to 10 days and then single or multiple lesions evolve, which arise mostly in the hands and face. At the onset of disease, primary lesions are the papules that gradually progress to nodular patterns. The nodules change into tubercule or crusted form within 4-6 weeks (5). The lesions may complicate to lymphangitis or secondary bacterial infection but systemic complications such as erythema multiforme, maculopapular rash, and generalized lymphadenopathy are rare (6, 7). In this paper, we present two cases of erythema multiforme following Orf disease.

## Case report:

***Case 1***

**Figure 1:**
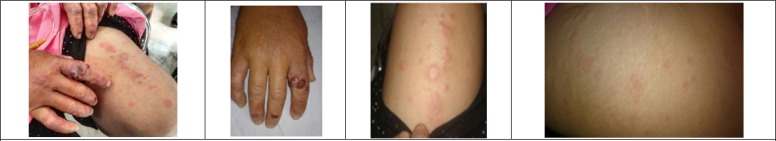
Skin lesions of case 1

A 45-year-old woman was admitted to the emergency department with chief complaint of generalized erythema, low-grade fever, and mild itching. She had a history of hypertension and captopril consumption from 3 years ago. On arrival, vital signs were stable and only a low fever was detected. On physical examination, disseminated maculopapular rash and target lesions in favor of erythema multiforme were seen. Moreover, there were several purple nodules with a brief fluctuation in the proximal phalange of right index finger and distal phalange of third right finger (figure 1). Finger lesions appeared 5 days after contact with a sheep and gradually enlarged during the 25 days before present complaint. Laboratory data showed mild leukocytosis. Based on clinical features, history of contact with sheep, and the high prevalence of disease in Mazandaran province, Iran, diagnosis of Orf disease was made with a high pretest probability for finger lesions, which is complicated to erythema multiforme. Patient was treated with warm compress, and low dose of intravenous corticosteroid and antihistamine. The generalized eruptions disappeared within five days with complete recovery after 6 weeks. 

***Case 2***

**Figure 2:**
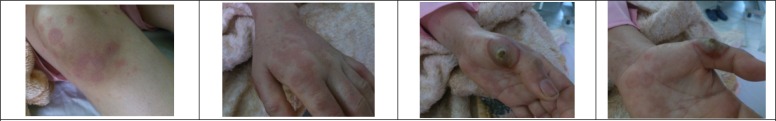
Skin lesions of case 2

The second case was a 32-year-old woman admitted to the emergency department with generalized maculopapular rash, low fever and sore throat. She had a negative medical and drug history. On arrival, vital signs were stable and physical examination revealed papulopustular lesions on first phalange of right thumb and generalized maculopapular rash with target lesions (figure 2). Patient remembered exposure to the head of a sheep several days ago. Same as the first presented case, erythema multiforme following Orf disease was diagnosed and supportive therapy with low doses of intravenous corticosteroid and antihistamine was administered. The systemic eruptions were cured within 6 days and Orf lesions disappeared after 5 weeks.

## Discussions:

Orf or pustular dermatitis or milker’s nodule is a disease caused by parapaxvirus transmitted to humans via contact with infected goat and sheep (8). The disease occurs most frequently on the fingers, hands and face. After an incubation period of 4-6 days, an erythematous papule appears and possibly goes to nodular and pustular form (9). Milker’s nodule can complicate to fever, lymphangitis, lymphadenopathy or bacterial super-infection. Rare complications of the disease are vesioculo-pustular eruptions such as erythema multiforme (9, 10). These reactions are considered to be an immune response to the Orf infection (10). Differential diagnoses include pyoderma, herpetic whitlow, cowpox, cat-scratch disease, anthrax, tularemia, tuberculosis, other mycobacteria, syphilis, sportrichosis, keratoacanthoma, and pyogenic granuloma (10). Diagnosis of Orf is usually based on clinical findings and history of exposure of non-intact skin to contaminated sheep and goat saliva and their dead body. Virus isolation, tissue culture and polymerase chain reaction (PCR) in some cases could be helpful but they are expensive and difficult (9, 10). Orf is a self-limiting disease and is completely resolved in about 4-6 weeks (2, 10). Conservative therapy and local antiseptic to prevent bacterial supper-infection are recommend, but for large lesions cryotherapy or topical cidofovir cream could be used (2, 11). Low dose systemic steroid and antihistamines are useful in treatment of erythema multiforme (12).

## Conclusion:

Orf disease should be considered as a possible underlying cause of erythema multiforme in endemic area. 
